# Efficacy of new intermittent abdominal pressure ventilator for post-ischemic cervical myelopathy ventilatory insufficiency

**DOI:** 10.1186/s40248-019-0169-4

**Published:** 2019-01-28

**Authors:** Paolo I. Banfi, Eleonora Volpato, John R. Bach

**Affiliations:** 1 IRCCS Santa Maria Nascente, Fondazione Don Carlo Gnocchi, Milan, Italy; 2 Department of Psychology, Università Cattolica del Sacro Cuore, Milan, Italy; 3 Department of Physical Medicine and Rehabilitation, Rutgers University New Jersey Medical School, Newark, USA

**Keywords:** Non-invasive ventilation (NIV), Tracheostomy mechanical ventilation (TMV), Intermittent abdominal pressure ventilator (IAPV), Quality of life (QoL)

## Abstract

Non-invasive ventilation (NIV) is the treatment of choice for patients symptomatic for respiratory muscle dysfunction. It can normalize gas exchange and provide up to continuous non-invasive ventilator support (CNVS) as an alternative to intubation and tracheotomy. It is usually provided via non-invasive facial interfaces or mouthpieces, but these can be uncomfortable and uncosmetic. The intermittent abdominal pressure ventilator (IAPV) has been used for diurnal ventilatory support since 1938 but has been off the market since about 1990. Now, however, with greater emphasis on non-invasive management, a new IAPV is available. A patient with chronic ventilatory insufficiency post-ischemic cervical myelopathy, dependent on sleep NVS since 2003, developed symptomatic daytime hypercapnia for which he also used diurnal NVS via nasal pillows. However, he preferred not having to use facial interfaces. When not using diurnal NVS he was becoming dyspnoeic. Diurnal use of an IAPV was introduced. Arterial blood gas analysis using the IAPV decreased his blood pH from 7.45 to 7.42, PaCO_2_ from 58 to 37 mmHg, and improved PaO_2_ from 62 to 92 mmHg. At discharge, the patient used the IAPV 8 h/day with improved mood and quality of life. Consequently, he returned to work as a painter.

## Introduction

Non-invasive ventilation or “NIV” has come to refer to continuous positive airway pressure (CPAP) or bi-level PAP used at setting inadequate for full non-invasive ventilator support (NVS). However, non-invasive positive pressure ventilation is increasingly being used for continuous NVS (CNVS) as an alternative to tracheostomy mechanical ventilation (TMV) for patients with ventilatory pump failure. Over 1000 CNVS users have been described [1–4]. It improves gas exchange, symptoms [5], quality of life [6], decreases the incidence of pneumonia [7], and can be used to avert need for intubation and tracheotomy [8]. Whereas tracheostomy tends to increase ventilator dependence, detracts from quality of life, and is associated with reactive depression, NIV/NVS facilitates ventilator weaning and extubation [9, 10]. It is delivered via facial interfaces including nasal, oronasal, nasal prongs, and mouthpieces for diurnal and sleep ventilatory assistance/support [3]. However, the interfaces can cause skin discomfort and, at times, ulcerations, airway dryness and congestion and can impact negatively on quality of life and gas-exchange [11–13]. Moreover, oxyhemoglobin desaturations and dyspnea can occur with interface disconnection from NVS [11].

**Fig. 1:**
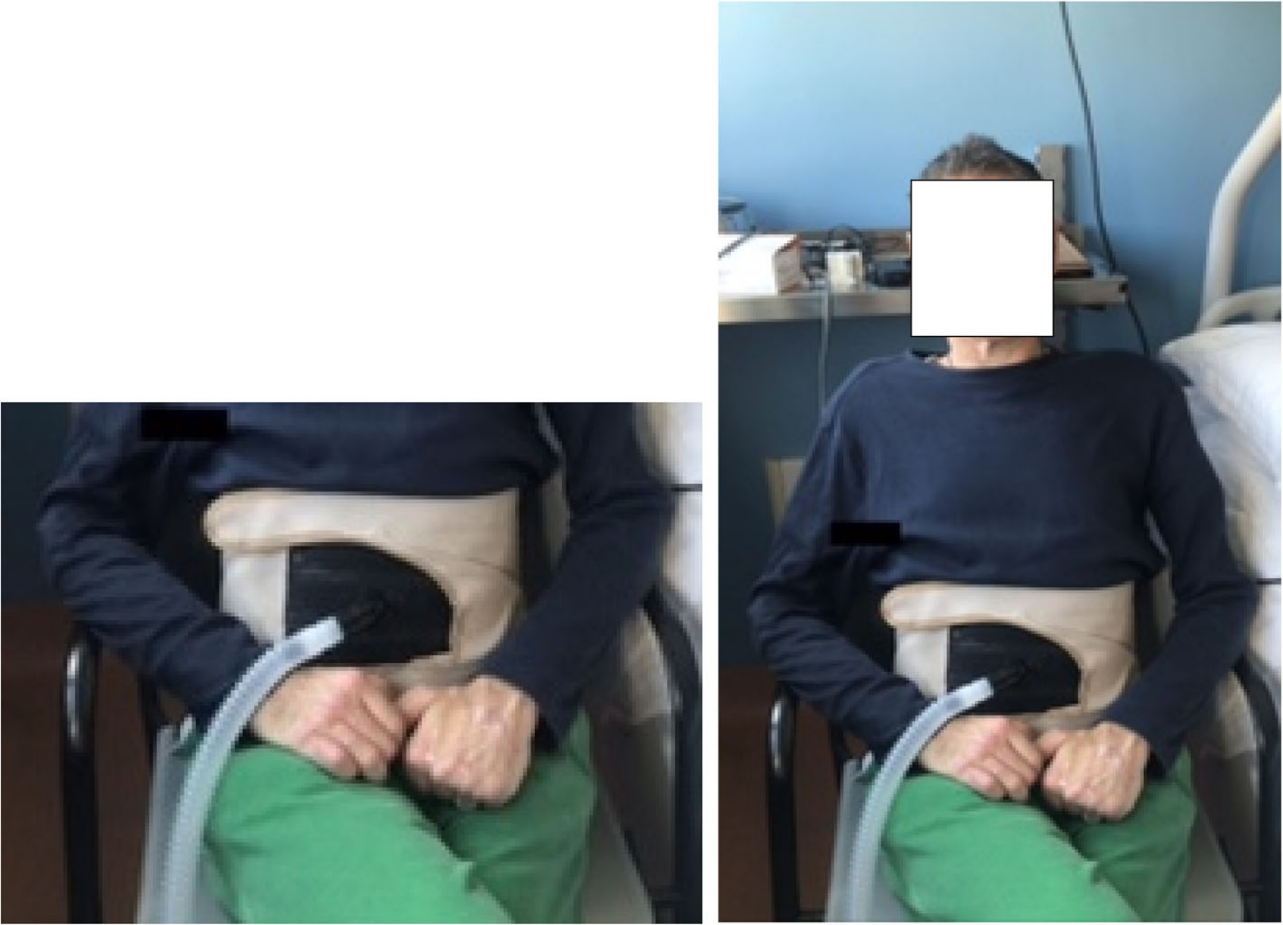
The PBAir™ corset and air bladder of the new IAPV

The LUNA DS (Dima Italia Inc., Bologna, Italy) is a portable ventilator, that along with the PBAir™ corset is an easy to use IAPV. The LUNA has an internal battery capacity that can also be used for NVS for sleep. It provides a dedicated IAPV ventilation mode. The IAPV corset is lightweight (Fig. 1), comfortable, and easy to don and fit with Velcro fasteners. Like earlier IAPVs, cyclical inflation of a rubber bladder inside the corset moves the diaphragm upwards to expel air from residual volume. This causes air to enter the lungs via the upper airway as gravity draws the diaphragm back to its resting position. The IAPV eliminates need for facial ventilation interfaces.

The following IAPV parameters can be set: PBelt™ (pressure inside the bladder), Tinsp (real inspiratory time when the diaphragm moves down), frequency (respiratory rate), and Rise Time (time to inflate the bladder). The IAPV only works efficiently when a patient is in sitting position at an angle of 30° or greater and is optimal at 75° [14]. It optimizes cosmesis, facilitates social interaction and communication, leaves the field of vision free, and allows the patient a more normal sense of smell by eliminating facial interfaces that are colonized by often pathogenic bacteria, thereby favouring oral nutrition [15, 16].

## Case report

A 51-year-old male painter with a 40 pack/years history of cigarette smoking and a diagnosis of chronic respiratory failure due to post ischemic cervical myelopathy was trained in bi-level PAP which he used up to 16 h per day since 2003 at 25 inspiratory cm H_2_O (IPAP) and 7 cm H_2_O expiratory (EPAP) pressures and rate 15/min. However, during the last year he experienced daytime dyspnoea, tachypnoea, orthopnea, and deterioration of gas exchange when not using it and complained of the interface causing discomfort and interfering with his daytime employment. His arterial blood gases (ABG) breathing unassisted in ambient air 4 h after discontinuing nocturnal bi-level PAP was PaO_2_ 62 and PaCO_2_ 58 mmHg. On admission, he was placed on IAPV ventilation. He wore the IAPV corset’s horizontal upper border two fingers below the costophrenic junction.

His spontaneous tidal volumes of 172–180 ml increased to 771–908 ml using the IAPV with the LUNA ventilator set at 24 cm H_2_O pressure, rate 15/min (Table 1). Arterial blood gases were monitored after the second hour of IAPV use. Table 2 demonstrates normalization of the diurnal breathing pattern and gas exchange. After 3 months his PaO_2_ breathing unassisted in ambient air was 75 and PaCO_2_ 44 mmHg (Table 2).

**Table 1:** Respiratory assessment: a comparison between spontaneous breathing and the intermittent abdominal pressure ventilator

	Spontaneous Breathing		IAPV/PBAir	
	Min	Max	Min	Max
Frequency	25.4 cpm	35.3 cpm	14.2 cpm	14.2 cpm
Inspiratory Volume	172 mL	280 mL	771 mL	908 mL
Expiratory Volume	165 mL	277 mL	791 mL	923 mL
Flow	−32.2 Llpm	+ 33.5 lpm	−61.9 lpm	+ 56.1 lpm

**Table 2:** Pulmonary gas exchange

	Baseline SB	After 2 h during IAPV	1 month SB	3 months SB
Ph	7.45	7.42	7.45	7.42
PaO_2_	62	92	71	75
PaCO_2_	58	37	48	44
HCO_3_-/EB	31.5/4.9	28.2/1.9	27.4/2.6	25.2/0.9

*ABG* Arterial Blood Gas Analysis, *SB* Spontaneous breathing, *IAPV* Intermittent Abdominal Pressure Ventilation

Quality of life parameters were measured and at discharge thanks to the EuroQoL (EQ-5D) [17] and the World Health Organization Quality of Life Questionnaire (WHOQOL-Bref) [18], the patient used the IAPV 8 h/day with improved mood (assessed by the Hospital Anxiety and Depression Scale (HADS) [19]) and cognition (as assessed by the Mini Mental Status Examination [20] and the Addenbrooke’s Cognitive Examination Revised (ACE-R) [21] (Table 3). Moreover, three months later he reported that the IAPV was still effectively relieving his former daytime dyspnoea but that he had achieved up to 6 h/d of autonomous breathing without dyspnoea or tachypnea.

**Table 3:** Principal data of the psychological evaluation pre- and post- use of the IAPV

Psychological Evaluation						
Age, years:	51					
Profession:	Artist					
Manual predominance:	Dx					
Test	Admission			Discharge		
	Raw Score	Adjusted Score	Equivalent Score	Raw Score	Adjusted Score	Equivalent Score
Spatial orientation [20]:	5/5			5/5		
Time orientation [20]:	5/5			5/5		
Mini Mental State Examination (MMSE) [20]:	27		*Non Case*	29		*Non Case*
Addenbroke’s Examination (ACE-R) [21]:	86/100	84.89	3	88/100	86.89	3
EuroQoL (EQ-5D) [17]:	31211			21111		
Index EQ-5D [17]:	0.3			0.85		
Visual Analogue Scale (VAS EQ-5D) [17]:	60			100		
World Health Organization Quality of Life Questionnaire-Bref (WHOQoL-Bref) [18]:	92			109		
Physical Domain:	10.66	41.66		19.33	95.83	
Psychological Domain:	20	100		21.6	110	
Environment Domain:	20	100		20.66	104.16	
Social Domain:	26	137.5		28	150	
Hospital Anxiety and Depression Scale (HADS) [19]:	25/42			5/42		
HAD-A:	11/21		*Moderate*	4/21		*Non Case*
HAD-D:	7/21		*Non Case*	1/21		*Non Case*

## Discussion

C. J. McSweeney described the Bragg-Paul Pulsator, a IAPV that was used for 34 patients with acute diphtheritic respiratory muscle paralysis in 1938 [22–24]. The IAPV was perfected by Dr. Alvin Barach and his engineer William Smith in the 1940s [23]. Until the 1990s it was used in combination with NVS modalities instead of tracheostomy mechanical ventilation (TMV). However, with the treatment paradigm shift to TMV in the 1960s, the IAPV went off the market and there have been no major publications of patients using it since 1991 [22]. Now, however, with the paradigm shifting back to non-invasive management and over the last decade over 1500 750 CNVS users described [1–4], the IAPV is back on the European market [12, 13, 25, 26] and consideration of this practical, convenient, and comfortable daytime ventilation alternative is warranted. IAPV use had been limited by the relative lack of portability and inconvenience of formerly available large and heavy powerful ventilators needed to operate them, and the fact that clothing could catch on the corset buckles. Use of the IAPV had also never been reported by patients with cervical myelopathies. In our patient, the IAPV improved blood gases, relieved dyspnoea, and continued to be a comfortable alternative to bi-level PAP.

## Conclusion

This case of IAPV use for ventilatory assistance for a patient with a post-ischemic cervical myelopathy suggests that it can be a safe and comfortable alternative to daytime NIV and TMV leading to a better quality of life for this patient population.

## Abbreviations

ABG: Arterial Blood Gases
CNVS: Continuous Non-Invasive Ventilatory Support
CPAP: Continuous Positive Airway Pressure
EPAP: Expiratory Positive Air Pressure
IAPV: Intermittent Abdominal Pressure Ventilator
IPAP: Inspiratory Positive Air Pressure
NIV: Non-invasive Ventilation
NVS: Non-Invasive Ventilator Support
TMV: Tracheostomy Mechanical Ventilation
